# Low Frequency of Circulating CD8^+^ T Stem Cell Memory Cells in Chronic Chagasic Patients with Severe Forms of the Disease

**DOI:** 10.1371/journal.pntd.0003432

**Published:** 2015-01-08

**Authors:** Jose Mateus, Paola Lasso, Paula Pavia, Fernando Rosas, Nubia Roa, Carlos Andrés Valencia-Hernández, John Mario González, Concepción J. Puerta, Adriana Cuéllar

**Affiliations:** 1 Grupo Inmunobiología y Biología Celular, Pontificia Universidad Javeriana, Bogotá, Colombia; 2 Laboratorio de Parasitología Molecular, Facultad de Ciencias, Pontificia Universidad Javeriana, Bogotá, Colombia; 3 Fundación Clínica Abood Shaio, Bogotá, Colombia; 4 Facultad de Medicina, Pontificia Universidad Javeriana, Bogotá, Colombia; 5 Laboratorio de Parasitología, Instituto Nacional de Salud, Bogotá, Colombia; 6 Grupo de Ciencias Básicas Médicas, Facultad de Medicina, Universidad de los Andes, Bogotá, Colombia; Federal University of São Paulo, Brazil

## Abstract

**Background:**

CD8+ T cells have been shown to play a crucial role in *Trypanosoma cruzi* infection. Memory CD8+ T cells can be categorised based on their distinct differentiation stages and functional activities as follows: stem cell memory (TSCM), central memory (TCM), transitional memory (TTM), effector memory (TEM) and terminal effector (TTE) cells. Currently, the immune mechanisms that control *T. cruzi* in the chronic phase of the infection are unknown.

**Methodology/Principal Findings:**

To characterise the CD8+ T cell subsets that could be participating in the control of *T. cruzi* infection, in this study, we compared total and *T. cruzi*-specific circulating CD8+ T cells with distinctive phenotypic and functional features in chronic chagasic patients (CCPs) with different degrees of cardiac dysfunction. We observed a decreased frequency of total TSCM along with an increased frequency of TTE in CCPs with severe disease. Antigen-specific TSCM cells were not detectable in CCPs with severe forms of the disease. A functional profile of CD8+ T cell subsets among CCPs revealed a high frequency of monofunctional CD8+ T cells in the most severe patients with IFN-γ+- or TNF-α+-producing cells.

**Conclusions/Significance:**

These findings suggest that CD8+ TSCM cells may be associated with the immune response to *T. cruzi* and outcome of Chagas disease, given that these cells may be involved in repopulating the T cell pool that controls infection.

## Author Summary

Chagas disease is caused by the intracellular parasite *Trypanosoma cruzi*. After the onset of acute infection, all individuals enter the chronic phase and approximately 70% of them never have symptoms. However, nearly 30% of infected individuals develop symptoms, mainly of heart disease, even decades after the initial infection. Currently, it is unclear how the immune response controls infection and prevents the development of heart disease in some infected people. We have characterised the memory CD8^+^ T cell subsets in chronic chagasic patients, including a newly described population of cells called memory stem cells. This T cell subset seems important for replenishing the other T cell populations. The findings in this manuscript show that chronic chagasic patients with severe disease have the following: a) a low frequency of memory stem cells, b) no antigen-specific memory stem cells, and c) CD8^+^ T cells with less effector function compared with asymptomatic patients. These results indicate that the lack of T cell population renewal and the decrease in cells with multiple effector functions may be associated with the clinical outcome of chronic Chagas disease.

## Introduction

The memory CD8^+^ T cell compartment comprises cells that represent distinct differentiation stages and different functional activities. This cellular compartment has been divided into stem cell memory (T_SCM_), central memory (T_CM_), transitional memory (T_TM_), effector memory (T_EM_) and terminal effector (T_TE_) cells [1]. T_SCM_ are considered an early differentiated and long-lived human memory T cell population with an enhanced capacity for self-renewal and a multipotent ability to generate other subsets of memory cells [2]. As shown in a viral infection model in non-human primates, T_SCM_ cells also demonstrate better survival capacity compared with conventional memory T cells, even in the presence of little or no antigen stimulus [3]. Therefore, as a potential reservoir to maintain and to replenish the memory T cell pool, T_SCM_ cells represent a potential tool for cellular immune therapies in chronic infectious diseases.

Chagas disease (CD), caused by the intracellular parasite *Trypanosoma cruzi*, is a public health problem that affects nearly 8 million people in Latin America, and almost 25 million people are at risk for contracting this disease [4]. In addition, cases are reported on different continents due to the migration of people from CD-endemic countries [5]. Classically, the course of CD consists of consecutive acute and chronic phases. The acute phase, lasting several weeks, is associated with a high parasitaemia that can be controlled but not eliminated by the immune system. Indeed, parasite persistence at low levels is the hallmark of the indeterminate or asymptomatic phase, which can last a lifetime. However, between 30–40% of infected individuals in the chronic phase develop a symptomatic phase with heart or gastrointestinal involvement [6].

Currently, the immune mechanisms that control *T. cruzi* infection and do not permit chronic phase progression are unknown. However, in mouse models of *T. cruzi* infection, it was shown that CD8^+^ T cells contribute to the control of intracellular pathogen infection by secreting cytokines and perforin. For example, CD8^+^ T cell knockout (KO), IFN-γ KO and perforin KO mice infected with *T. cruzi* were unable to control parasitemia and succumbed faster to infection than wild-type infected mice [7], [8]. In humans with severe cardiac forms of CD, it has been demonstrated that CD8^+^ T cells decline both in number and function, and there is a low frequency of early differentiated cells along with a high frequency of late differentiated cells compared with patients with less severe forms of the disease [9]. Additionally, patients with severe disease forms have a lower frequency of IFN-γ-producing T cells than patients with mild forms [9], [10]. Indeed, a low frequency of IFN-γ-producing CD4^+^CD8^+^ T cells, reduced proliferative capacity and CD28 expression in T cells have been observed in patients with severe forms of the disease in previous group studies [11], [12]. As CD8^+^ T cells are a heterogeneous population with distinct proliferative, survival and functional abilities, it is important to characterise CD8^+^ T cell subsets in chronic chagasic patients (CCPs) to define the types of cellular immune responses participating in the control of *T. cruzi*. The aim of the present study was to compare circulating CD8^+^ T cell subsets in CCPs with different degrees of disease severity, with particular focus on T_SCM_ cells, which have the capability to generate all memory subsets.

## Methods

### Ethics statement

The Research and Ethics Committees from the Pontificia Universidad Javeriana, Instituto Nacional de Salud, Fundación Abood Clínica Shaio and Hospital Universitario San Ignacio approved this study following the national regulations and the Declaration of Helsinki. Signed informed consent was obtained from all individuals prior to their inclusion in the study.

### Study participants

A total of 32 cardiac CCPs from endemic areas were recruited at the Instituto Nacional de Salud, Fundación Abood Clínica Shaio and Hospital Universitario San Ignacio in Bogotá, Colombia. Additionally, nine healthy donors (HD) from non-endemic areas were included. All subjects were tested for *Trypanosoma cruzi* antibodies using an indirect immunofluorescence assay (IFI) and an enzyme-linked immunosorbent assay (ELISA). CCPs were classified into groups A, B, C or D according to their disease severity score as previously described [13]. Group A included individuals with a normal electrocardiogram (ECG), heart size and left ventricular ejection fraction (LVEF) and a New York Heart Association (NYHA) class I designation. Group B individuals had an abnormal ECG but normal heart size and LVEF and a NYHA class I designation. Group C individuals had an abnormal ECG, increased heart size, reduced LVEF and a NYHA class II or III designation. Finally, group D individuals had an abnormal ECG, increased heart size, reduced LVEF and were NYHA class IV. Patients from groups A and B correspond to patients with mild forms of disease severity, and those from groups C and D are patients with severe forms. Clinical characteristics and the classification of study participants are reported in Table 1.

**Table 1:** Characteristics of study participants.

Variable	CCPs#				HDs	*p* value
Subjects, n	32				9	-
Age (years), median (range)	55 (26–80)				45 (32–60)	0.1029+
Female (%)	59.37				22.22	-
	A	B	C	D		
Patients, n (%)	10 (31.25)	8 (25.00)	9 (28.12)	5 (15.63)	-	-
Age (years), median (range)	43 (26–64)###	50 (38–67)#	70 (55–80)	65 (45–76)	-	0.0005++
LVEF, median (range)	60 (50–65)	60 (48–65)	30 (15–50)*	20 (10–35)**	-	0.0001++

CCPs, chronic chagasic patients; HDs, healthy donors; LVEF, left ventricular ejection fraction.

# Clinical characteristics of CCPs are described in Materials and Methods.

+ Mann-Whitney test, CCP svs. HDs.

++ One-way ANOVA non-parametric Kruskal–Wallis test with Dunn's test.

*p<0.05 (A vs. C, B vs. C),

**p<0.001 (A vs. D, B vs. D).

### *p*<0.0001 (A vs. C),

# *p*<0.05 (B vs. C).

### Blood samples

Blood samples were obtained from all study participants in EDTA and heparinised tubes (BD Vacutainer; Franklin Lakes; NJ, USA). The absolute number of lymphocytes was determined from the EDTA tube by a standard differential blood count. Peripheral blood mononuclear cells (PBMCs) were isolated with a Ficoll-Hypaque density gradient (GE Healthcare; Uppsala, Sweden) from the heparinised tubes. Non-frozen cells were used in phenotypic and functional activity analyses.

### Antibodies

The following conjugated antibodies were used for cell-surface staining: CD3-Pacific Blue (BD Pharmingen; Clone UCHT1; Cat. No. 558117; San Diego, CA, USA), CD8-APC H7 (BD Pharmingen; Clone SK1; Cat. No. 641400), CD45RA-PE (BD Pharmingen; Clone HI100; Cat. No. 555489), CCR7-PE-Cy7 (BD Pharmingen; Clone 3D12; Cat. No. 557648), CD28-PerCP-Cy5.5 (BD Biosciences; Clone L293; Cat. No. 337181; San Jose, CA, USA), CD27-Alexa Fluor 700 (BD Pharmingen; Clone M-T271; Cat. No. 560611), CD95-APC (BD Pharmingen; Clone DX2; Cat. No. 558814) and CD127-FITC (BD Pharmingen; Clone HIL-7R-M21; Cat. No. 560549). Conjugated antibodies for intracellular staining included the following: IFN-γ-FITC (BD Pharmingen; Clone 4S.B3; Cat. No. 554551), IL-2-PerCP-Cy5.5 (BD Pharmingen; Clone MQ1-17H12; Cat. No. 560708) and TNF-α-AlexaFluor 700 (BD Pharmingen; Clone MAb11; Cat. No. 557996). To exclude dead cells, the Fixable Aqua Dead Cell Stain viability marker was used (Invitrogen; Cat. No. L34957; Eugene, OR, USA).

### Cell-surface phenotypic and intracellular cytokine staining using flow cytometry

All conjugated antibodies were titrated, and each multicolour panel of conjugates was evaluated as previously described [14]. To evaluate the frequency of CD8^+^ T cell subsets, one million PBMCs were stained with the viability marker for 20 min in the dark at room temperature and then washed with PBS 0.001 M pH 7.4 (1X PBS) (Eurobio; Les Ulis, France). Cells were stained with antibodies against CD3, CD8, CD45RA, CCR7, CD28, CD27, CD127 and CD95 molecules for 30 min in the dark at 4°C and washed with 1X PBS. To evaluate the cytokine production of CD8^+^ T cell subsets, one million PBMCs were cultured with anti-CD28 and anti-CD49d antibodies and incubated for 6 hours in the presence of brefeldin A (BD Biosciences, San Jose, CA, USA) with Staphylococcal enterotoxin B (SEB) (Sigma-Aldrich; Saint Louis, MO, USA), *Trypanosoma cruzi* trypomastigote lysate or medium. Parasite lysate was obtained as previously described [14]. First, cells were stained with the viability marker and then with surface antibodies against CD3, CD8, CD45RA, CCR7 and CD95 molecules for 30 min in the dark at 4°C. Cells were washed with 1X PBS, fixed and permeabilised with Cytofix/Cytoperm (BD Biosciences) for staining with antibodies against IFN-γ, TNF-αand IL-2 for 30 min in the dark at 4°C, followed by washing with 1X Perm/Wash (BD Biosciences). At least 50,000 events gated on CD3^+^CD8^+^ cells were acquired on a FACS Aria II flow cytometer. Analysis was performed using FlowJo 9.3.2 (Tree Star; Ashland, OR, USA), Pestle 1.7 (National Institutes of Health (NIH), Bethesda, MD, USA) and SPICE 5.3 (NIH) software [15]. Dead and doublet cells were excluded from the analysis, as previously described [14]. A positive cytokine response was defined by subtracting the cytokine background (cells cultured with medium) from a frequency of >0.05% for each CD8^+^ T cell subset (average frequency of the response of CD8^+^ T cells from HDs cultured with parasite lysate plus 3 SD).

### PCR for parasite detection

Conventional PCR (cPCR) and quantitative PCR (qPCR) were used to assess parasite DNA in blood samples in guanidine hydrochloride-EDTA stored at 4°C from 30 CCPs. DNA from blood was extracted using a High Pure PCR template preparation kit (Roche, Mannheim, Germany). Afterwards, cPCR was run using initiators of β-globin FR, as described previously [16], to check DNA integrity and to rule out the presence of inhibitors in the sample. cPCR was performed with the S35 (AAATAATGTACGGG(T/G)GAGATGCATGA) and the S36 (GGGTTCGATTGGGGTTGGTGT) primers, which amplify the kDNA variable mini-circle region from *T. cruzi,* using PCR reaction conditions described previously [17], [18]. qPCR was performed with Cruzi 1 (ASTCGGCTGATCGTTTTCGA) and Cruzi 2 (AATTCCTCCAAGCAGCGGATA) primers and the Cruzi 3 (6FAM-CACACACTGGACACCAA-BBQ) probe, which amplify a 166-bp segment of the satellite DNA from *T. cruzi* [19]. Each sample was run in duplicate, and the parasite load was estimated based on a standard curve. The curve was constructed with different concentrations of genomic DNA mixed with blood from one uninfected donor ranging from 10^5^–10^0^ parasite equivalents per mL. Samples were run using a LightCycler 1.5 Instrument (Roche). For both PCR methods, different controls were included: reaction (water added in the room containing the reaction mixture), grey (water added in the room where the sample was added to the reaction), negative (genomic DNA from an HD) and positive (genomic DNA of *T. cruzi*).

### Statistical analysis

A statistical analysis was performed using the Mann-Whitney test or a one-way ANOVA non-parametric Kruskal–Wallis test with Dunn's test for multiple comparisons. Correlations between the frequencies and the absolute numbers of CD8^+^ T cell subsets were analysed using Spearman's rank correlation coefficient. A Wilcoxon signed-rank test was performed to compare stimulated cells with 2 functions or 1 function. All tests were two-tailed, and statistical significance was achieved with *p*<0.05. GraphPad Prism 6.0 for Mac OS X (San Diego, CA, USA) software was used for statistical analyses.

## Results

### Distribution of total CD8^+^ T cell subsets from CCPs

**Figure 1:**
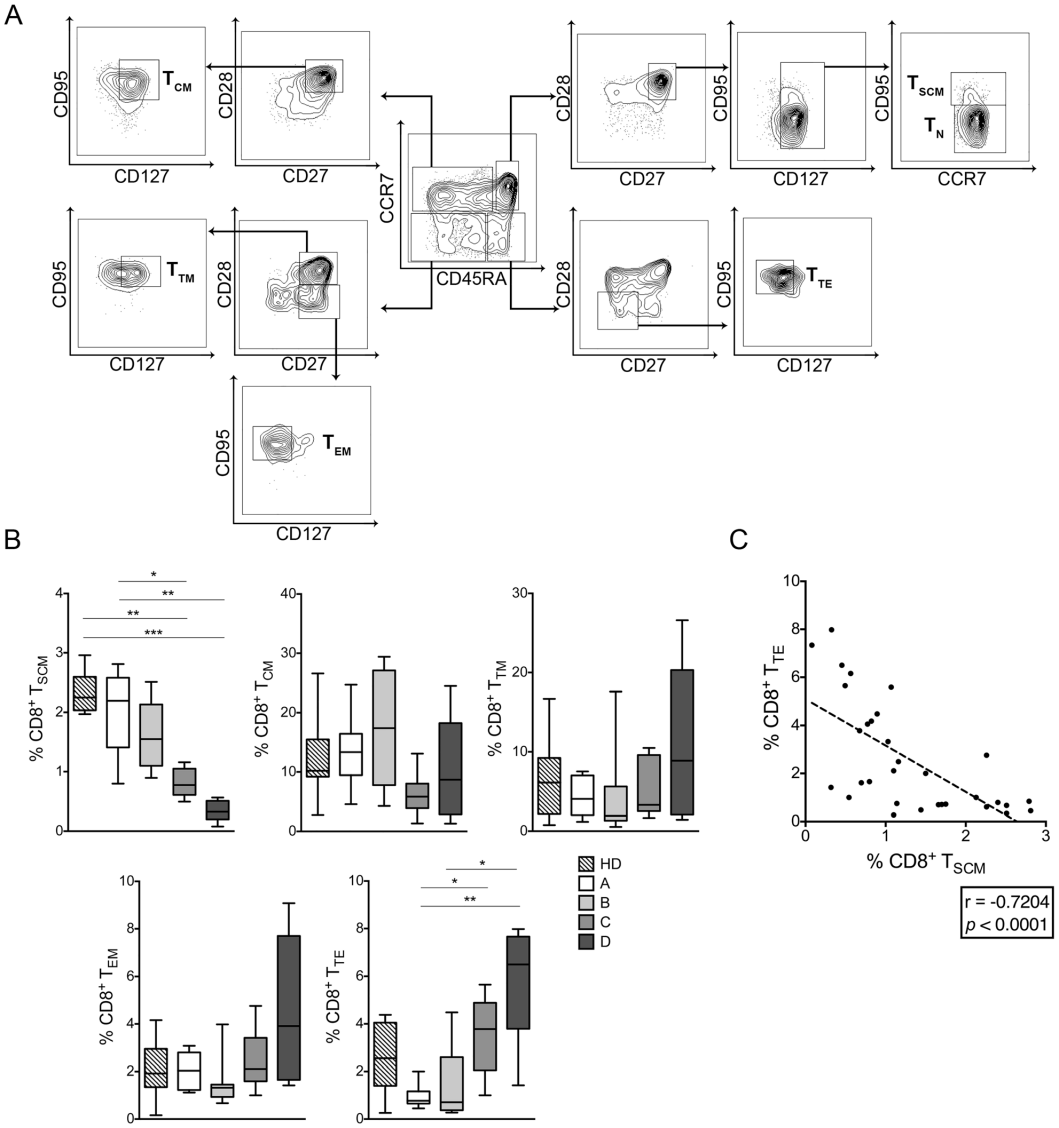
Distribution of total CD8^+^ T cell subsets from CCPs and HDs. (**A**) Representative contour plot of the ex vivo selection of CD8^+^ T cell subsets based on the differential expression of CD45RA, CCR7, CD28, CD27, CD95 and CD127. (**B**) Frequency of CD8^+^ T_SCM_, T_CM_, T_TM_, T_EM_ and T_TE_ cells from CCPs with different degrees of disease severity and HDs. Box and whiskers indicate the median frequency and range of the total CD8^+^ T cell subsets (25–75 percentile). The *p* values were calculated using a one-way ANOVA non-parametric Kruskal–Wallis test (*p<0.05, **p<0.01, ***p<0.001). (**C**) The correlations between the frequencies of CD8^+^ T_SCM_ cells and CD8^+^ T_TE_ cells from CCPs were calculated with Spearman's rank correlation coefficient. CCPs were grouped according to the disease severity as described in Materials and Methods (A = 10, B = 8, C = 9 and D = 5). Additionally, nine HDs were included.

T cells are a highly heterogeneous cell compartment comprising different phenotypes, functional activities, gene expression and survival capacities. Recently, CD45RA (or CD45R0), CCR7, CD28 and CD95 were proposed as canonical markers to identify T cell subsets using multiparametric flow cytometry [1]. In addition, CD127 and CD27 were included to accurately define T stem cell memory (T_SCM_) cells as described previously [2]. To compare the frequencies of total CD8^+^ T cell subsets among CCPs with different degrees of disease severity and HDs, PBMCs isolated from 32 CCPs and 9 HDs were labelled with a panel of conjugated antibodies as described in the Materials and Methods. Representative contour plots depicting the ex vivo selection of CD8^+^ T cell subsets based on differential expression of CD45RA, CCR7, CD28, CD27, CD95 and CD127 are shown in Fig. 1A. On the basis of previous data, CD8^+^ T cell subsets were defined as T_SCM_ cells (CD45RA^+^CCR7^+^CD28^+^CD27^+^CD95^+^CD127^+^), central memory (T_CM_) cells (CD45RA^−^CCR7^+^CD28^+^CD27^+^CD95^+^CD127^+^), transitional memory (T_TM_) cells (CD45RA^−^CCR7^−^CD28^+^CD27^+^CD95^+^CD127^+^), effector memory (T_EM_) cells (CD45RA^−^CCR7^−^CD28^−^CD27^+^CD95^+^CD127^−^) and terminal effector (T_TE_) cells (CD45RA^+^CCR7^−^CD28^−^CD27^−^CD95^+^CD127^−^) [1]. The frequencies of CD8^+^ T cell subsets were similar for group A and B patients and for HDs. A significant difference was observed when the frequencies of T_SCM_ and T_TE_ cells were compared between CCPs with severe and mild forms of the disease (Fig. 1B). No significant differences were observed for T_CM_, T_TM_ and T_EM_ frequencies between CCPs and HDs. Given that we found significant differences in the frequencies of T_SCM_ and T_TE_ cells from CCPs, we evaluated whether the reduced frequency of T_SCM_ cells was associated with changes in the frequency of T_TE_ cells. Indeed, in CCPs, the frequency of T_SCM_ cells correlated negatively with the frequency of T_TE_ cells (Spearman r = −0.7204, *p*<0.0001) (Fig. 1C). In addition, we found a negative trend in the frequency of T_SCM_ cells and a positive trend in the frequency of T_EM_ and T_TE_ cells in CCPs with various degrees of disease severity (S1 Fig.), as has been shown in previous reports [9]. As variations in the numbers of CD8^+^ T cells could consequently affect the absolute values of the studied subsets, absolute cell numbers were compared. The absolute numbers for CD8^+^ T cell subsets demonstrated a trend similar to that for the frequencies of CD8^+^ T cell subsets observed in CCPs and HDs. A correlation analysis of the absolute numbers of T_SCM_ and T_TE_ cells from all CCPs showed that T_SCM_ cells also correlated negatively with T_TE_ cells from CCPs (Spearman r = −0.4240, *p* = 0.0156, S2 Fig.).

### Distribution of *T. cruzi-*specific CD8^+^ T cell subsets from CCPs

**Figure 2:**
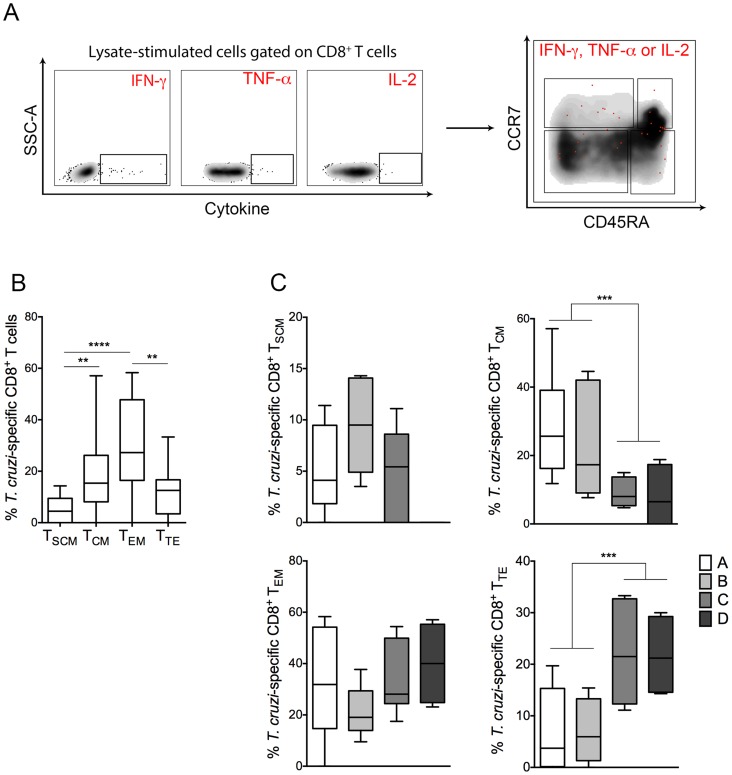
Distribution of *T. cruzi*-specific CD8^+^ T cell subsets from CCPs. (**A**) Representative density plot of the selection of *T. cruzi*-specific CD8^+^ T cells producing IFN-γ, TNF-α or IL-2 following cultivation with parasite lysate. A positive cytokine response was defined as described in Materials and Methods. (**B**) The proportions of *T. cruzi*-specific CD8^+^ T_SCM_, T_CM_, T_EM_ and T_TE_ cells were expressed as percentages of total cytokine-producing CD8^+^ T cells. The *p* values were calculated using a one-way ANOVA non-parametric Kruskal–Wallis test (**p<0.01, ****p<0.0001). (**C**) Frequencies of antigen-specific CD8^+^ T_SCM_, T_CM_, T_EM_ and T_TE_ cells among CCPs with differing degrees of disease severity. The *p* values were calculated using the Mann-Whitney test (***p<0.001). Box and whiskers indicate the median frequency and range of the *T. cruzi*-specific CD8^+^ T cell subsets (25–75 percentile). CCPs were grouped according to their disease severity as described in Materials and Methods (A = 8, B = 6, C = 6 and D = 4).

We next compared the frequencies of *T. cruzi*-specific CD8^+^ T cells bearing different T cell phenotypes in CCPs with various degrees of disease severity. To evaluate the frequency of antigen-specific CD8^+^ T cell subsets, PBMCs from CCPs were stimulated with parasite lysate and labelled with a panel of conjugated antibodies to assess the cytokine production of CD8^+^ T cell subsets. Assessment of antigen-specific CD8^+^ T cells was carried out for cytokine production in response to parasite lysate as described in the Materials and Methods. Representative density plots for the selection of *T. cruzi*-specific CD8^+^ T cells producing IFN-γ, TNF-αand IL-2 are shown in Fig. 2A. An increased frequency of antigen-specific T_EM_ cells was observed in all CCPs (Fig. 2B). CCPs with severe disease demonstrated a low frequency of antigen-specific T_CM_ cells and a high frequency of antigen-specific T_TE_ cells compared with patients with mild disease. Interestingly, we did not detect antigen-specific CD8^+^ T_SCM_ cells in patients from group D, who had the most severe form of the disease (Fig. 2C).

### Functional activity profiles of *T. cruzi*-specific CD8^+^ T cell subsets from CCPs

**Figure 3:**
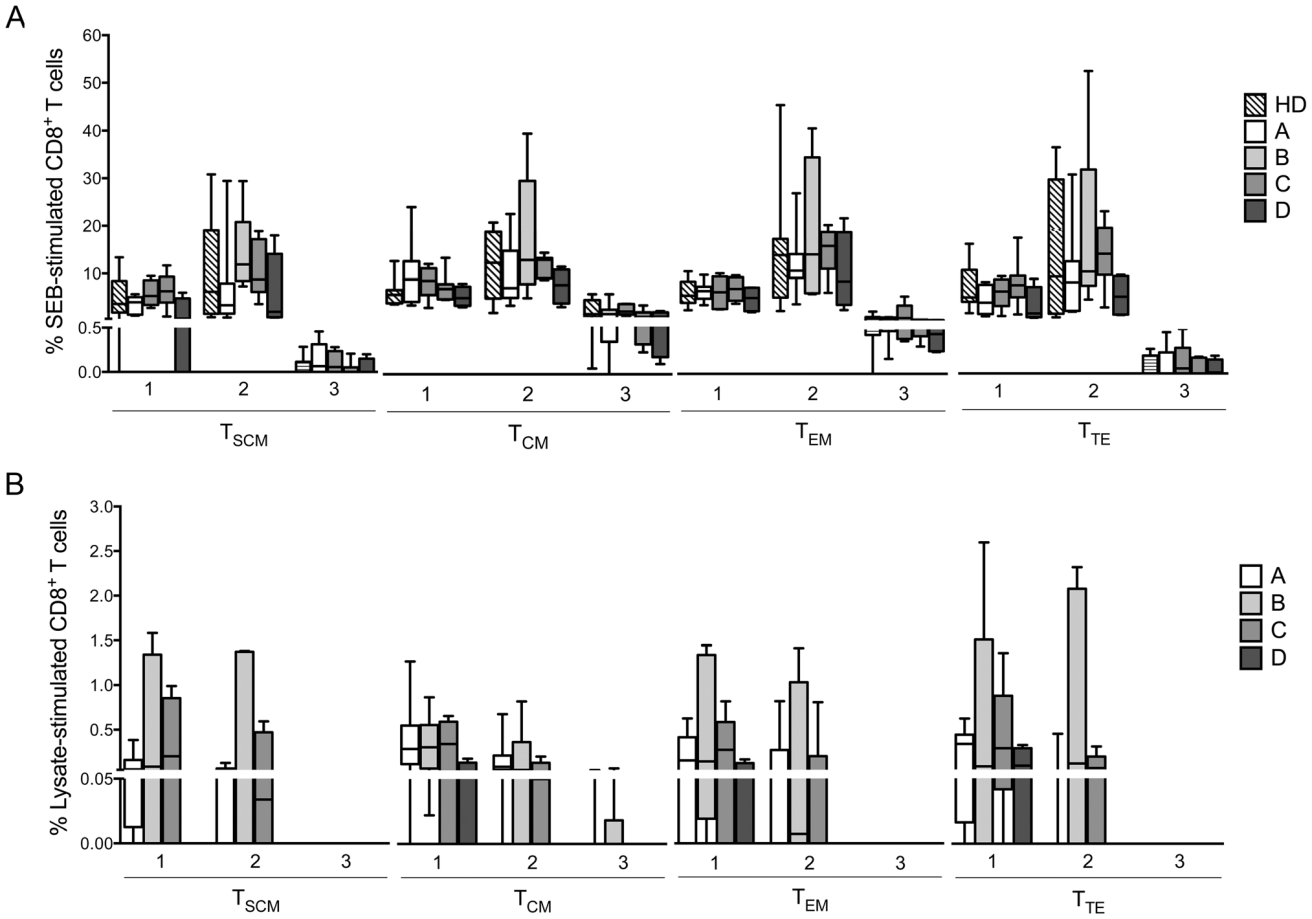
Functional activity profiles of *T. cruzi*-specific CD8^+^ T cell subsets from CCPs with different degrees of disease severity. (**A**) Frequencies of CD8^+^ T_SCM_, T_CM_, T_EM_ and T_TE_ cells with one, two and three functions among CD8^+^ T cells stimulated with Staphylococcal enterotoxin B (SEB). (**B**) Frequencies of CD8^+^ T_SCM_, T_CM_, T_EM_ and T_TE_ cells with one, two and three functions among CD8^+^ T cells stimulated with parasite lysate. Box and whiskers indicate the median frequency and range of the CD8^+^ T cell subsets (25–75 percentile). CCPs were grouped according to their disease severity as described in Materials and Methods (A = 8, B = 6, C = 6 and D = 4). Additionally, nine HDs were included.

**Figure 4:**
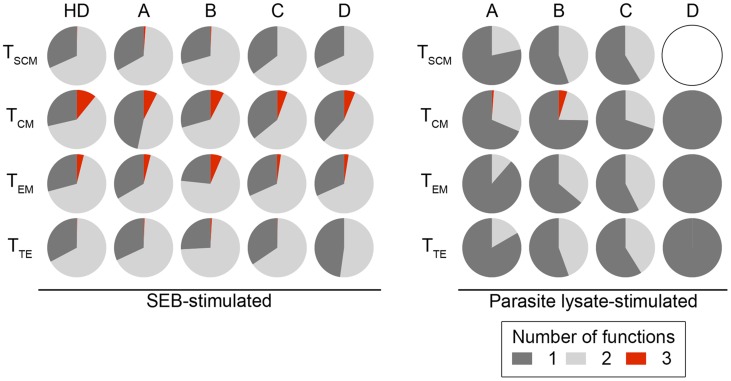
Proportions of functional activity profiles for *T. cruzi*-specific CD8^+^ T cell subsets from CCPs. Functional profiles of CD8^+^ T cell subsets stimulated with Staphylococcal enterotoxin B (SEB) and parasite lysate are color-coded according to the number of functions and summarised in the pie charts. Each pie slice corresponds to the mean production of one (dark grey), two (light grey) and three (red) functions. The white pie corresponds to the absence of a response when IFN-γ, TNF-α and IL-2 production were observed. CCPs were grouped according to their disease severity as described in Materials and Methods (A = 8, B = 6, C = 6 and D = 4).

**Figure 5:**
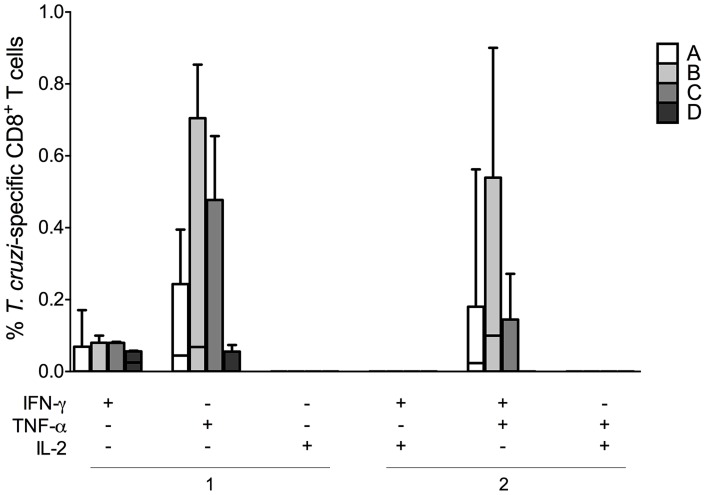
Cytokine combinations produced by *T. cruzi*-specific CD8^+^ T cells from CCPs. Frequencies of CD8^+^ T cells with one and two functions among CD8^+^ T cells stimulated with parasite lysate. Box and whiskers indicate the median frequency and range of the *T. cruzi*-specific CD8^+^ T cell subsets (25–75 percentile). CCPs were grouped according to their disease severity as described in Materials and Methods (A = 8, B = 6, C = 6 and D = 4).

Using the panel described above, we assessed the functional profiles of *T. cruzi*-specific CD8^+^ T cell subsets in CCPs and HDs in cultures with medium, SEB and parasite lysate. When comparing the frequencies of T_SCM_, T_CM_, T_EM_ and T_TE_ cells from CCPs with one or two functions in lysate-stimulated cells, we observed a high frequency of CD8^+^ T cell subsets with one function compared with cells with two functions (*p* = 0.0210, *p* = 0.0008, *p* = 0.0353 and *p* = 0.0006, respectively). However, among SEB-stimulated cells, there was a higher frequency of CD8^+^ T cell subsets with two functions than of cells with one function in all CCPs (S3 Fig.). As expected, in SEB-stimulated cells, we observed T_SCM_, T_CM_, T_EM_ and T_TE_ cells with three functions from CCPs in all groups, similar to those observed in HDs. In contrast, we observed an absence of cells with three functions among lysate-stimulated T_SCM_, T_EM_ and T_TE_ cells; however, patients from groups A and B had T_CM_ cells with three functions, which was not observed in patients from groups C and D. In lysate-stimulated cells in group D patients, there were no T_SCM_ cells with one, two or three functions among SEB-stimulated cells from both CCPs and HDs (Fig. 3). These findings were also observed in the proportion of cells because the patients from group D are composed of cells producing a single cytokine (Fig. 4). The most prevalent population in lysate-stimulated cells with two functions consisted of IFN-γ^+^TNF-α^+^-producing cells in CCPs with mild forms of the disease, and monofunctional cells were predominantly IFN-γ^+^ or TNF-α^+^ in patients from group D. Of note, IL-2-producing cells were not detected in any CCPs (Fig. 5).

To further investigate the associations between CD8^+^ T cell subsets and parasitaemia in CCPs, cPCR and qPCR were performed to assess the presence of parasites in peripheral blood as described in the Materials and Methods. Both PCR methods permitted the identification of 12 out of 30 (40%) CCPs with detectable parasitaemia (7 by cPCR and 9 by qPCR) (Table 2). Notably, we detected parasitaemia in 3 of 9 patients from group C; in contrast, in patients from groups A, B and D, the parasitaemia load was below the detection limit of qPCR. Due to the low numbers of individuals, there were no associations between CD8^+^ T cell subsets and parasitaemia; however, this finding demonstrated the presence of circulating parasites in all CCP groups, even those with the most severe forms of disease.

**Table 2:** CCPs with parasitaemia detectable by cPCR and qPCR.

PCR§	CCPs#			
	A	B	C+	D
Conventional	2/10	0/7	4/9	1/4
Quantitative	3/10	1/7	5/9	0/4
Total	5 (50%)	1 (14.3%)	5 (55.6%)	1 (25%)

CCPs, chronic chagasic patients.

§ The amplified PCR regions are described in Materials and Methods.

# Clinical characteristics of CCPs are described in Materials and Methods.

+ Only three of nine patients from group C had quantifiable parasite load in terms of parasite equivalents per mL; median (range): 3 (1–6).

## Discussion

Immunity aimed at antigen clearance or the control of disease progression has been shown to be directly related to the quality of the memory T cell response [20]. However, the study of memory T cells depends on technical approaches to describe the complex T cell compartment. In viral chronic infections, CD8^+^ T cell subsets have been extensively studied, but they have rarely been studied in infections caused by intracellular protozoans such as *T. cruzi*. In this study, we compared the total and antigen-specific circulating CD8^+^ T cell subsets among CCPs demonstrating different degrees of disease severity. Changes in the distribution of CD8^+^ T cell subsets could highlight the behaviour of cellular immunity during the natural history of infection and the pathogenesis of CD.

**Figure 6:**
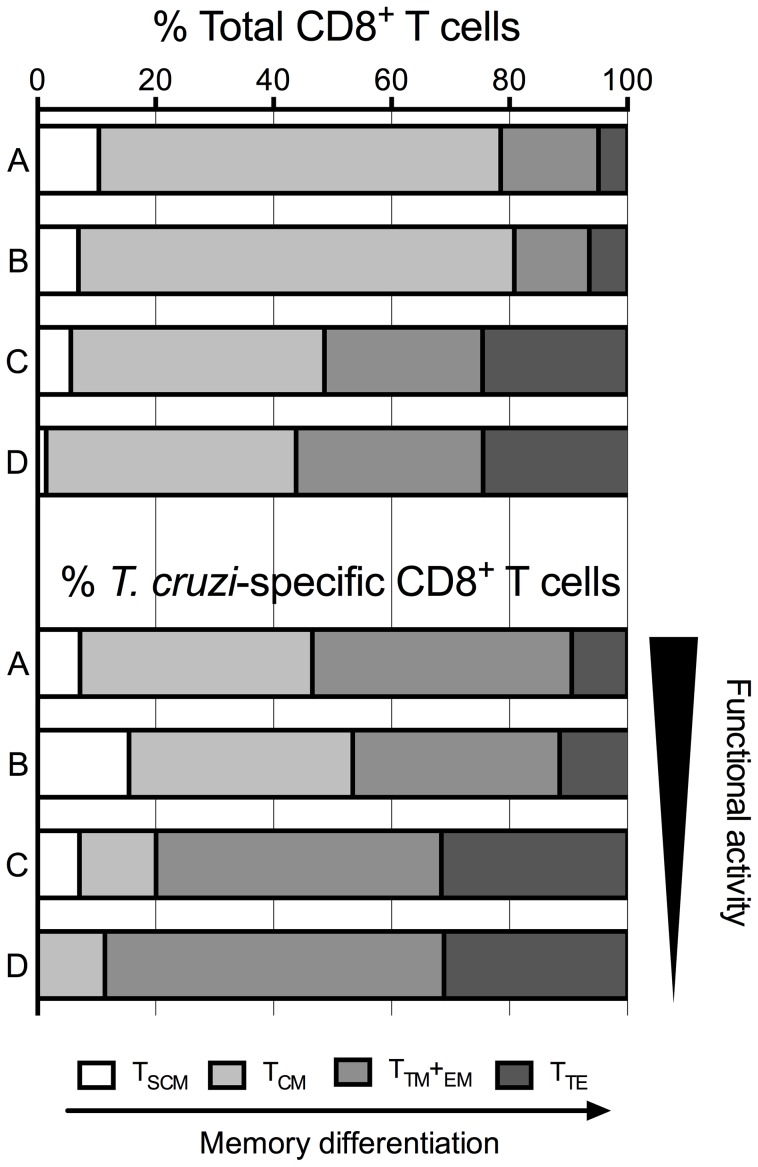
Proportions of total and *T. cruzi*-specific CD8^+^ T cell subsets from CCPs. Representation of the proportions of CD8^+^ T_SCM_, T_CM_, T_TM_+T_EM_ and T_TE_ cells from CCPs with different degrees of disease severity. The black bars represent the functional activities evaluated for parasite lysate-stimulated cells from CCPs for antigen-specific CD8^+^ T cell subsets.

We observed a decreased frequency in total T_SCM_ cells along with an increased frequency of T_TE_ cells in CCPs with severe forms of disease. Interestingly, IFN-γ-, TNF-α- and IL-2-producing antigen-specific T_SCM_ cells were not detectable in CCPs with severe forms of the disease. These changes observed for the T_SCM_ and T_TE_ subsets indicated a negative correlation both in the frequency and the absolute numbers of CD8^+^ T cells in all CCPs analysed. Conversely, when we studied the functional profiles of CD8^+^ T cell subsets among CCPs, a higher frequency of monofunctional antigen-specific CD8^+^ T cells was observed in CCPs with severe forms of disease. However, in SEB-stimulated cells, we observed cells with three functions among CD8^+^ T cell subsets from CCPs with all degrees of disease severity, similar to that observed in HDs (Fig. 6).

The T cell compartment is composed of different memory cells subsets, which are generated in response to antigen recognition. The recently identified T_SCM_ cells are characterised by their high proliferative and self-renewing capacities and their ability to differentiate into T_CM_ and T_EM_ cells [2]. In this study, we described the absence of antigen-specific T_SCM_ cells in CCPs with severe disease. Although T_SCM_ cells have not previously been described in CCPs, our findings are consistent with previous reports showing that the frequency of early differentiated CD8^+^ T cells decreases as the disease becomes more severe and the proportion of fully differentiated memory CD8^+^ T cells increases [9], [21]. Additionally, in a mouse model, it was observed that antigen-specific CD8^+^ T cells maintain a T_EM_ phenotype during persistent *T. cruzi* infection [22].

Recently, T_SCM_ cells have been associated with an improved prognosis in chronic HIV-infected patients because the frequency of CD8^+^ T_SCM_ cells decreased in all individuals with chronic HIV infection, but the frequency of these cells was restored in treated HIV-infected patients. Interestingly, these findings are in accordance with our results demonstrating that the frequency of CD8^+^ T_SCM_ cells decreased in CCPs with severe forms of disease [23]. In addition, T_SCM_ cells appeared to be progenitors for T_TE_ cells as these two compartments were inversely correlated. Taking into account that disease progression may last several years, it is difficult to know if this progress is due to the loss of T_SCM_ cells or if the loss of this subset is the result of a more severe infection. However, due to their capacities to generate all memory and effector T cell subsets, we hypothesized that the absence or a very low frequency of *T. cruzi*-specific T_SCM_ cells may be a contributing factor to failure to control the parasite during the symptomatic phase of the disease in *T. cruzi* chronic infection. In *Listeria monocytogenes*-infected mice, the adoptive transfer of cells expressing high amounts of IL-7R α-chain (CD127) helped to control infection due to the expansion of effector cell populations responsible for the rapid clearance of bacteria from the spleen and liver [24]. It is noteworthy that T_SCM_ cells express high levels of CD127 and other molecules associated with early differentiation [2]. The adoptive transfer of T_SCM_ cells in murine tumour models was shown to mediate potent tumour regression even better than T_CM_ and T_EM_ cells, and thus it has been proposed that these cells may be used for adoptive immunotherapy [2], [25]. However, T_SCM_ cell adoptive transfer has not been studied in chronic infections; thus, it would be interesting to evaluate the role of this cell population in the outcome of chronic infection.

In the present study, we included healthy donors from non-endemic areas as uninfected controls. However, it may be a different scenario when compared with healthy individuals exposed to *T. cruzi* in areas of endemic Chagas disease, who can have *T. cruzi*-specific T cells capable of producing IFN-γ even with negative conventional antibody testing for *T. cruzi* [26]. It is possible that repetitive exposure to *T. cruzi* in endemic areas may provide a persistent source of antigens that affect the proportion or function of CD8^+^ memory T cell subsets in healthy individuals, or some of the immune responses elicited by *T. cruzi* antigens in vitro may be induced by other protozoan parasites circulating in endemic areas [27], [28].

The polyfunctionality of CD8^+^ T cells has been proposed to be an immune correlate of protection in viral chronic infections [20]. Non-progressor patients chronically infected with human immunodeficiency virus (HIV) demonstrated a high frequency of polyfunctional CD8^+^ T cells compared with progressor patients [29]. Antigenic persistence in chronic infections is implicated in the impaired cellular immune response due to excessive activation of the immune system [30]. In the peripheral blood of CCPs, a high frequency of T_TE_ cells in patients with severe disease and a low frequency or absence of antigen-specific cells with one, two or three functions as assessed by cytokine production were observed. This phenomenon could be attributable to the high frequency of cells with a late differentiated stage, as these cells have decreased polyfunctional capacities. Indeed, we observed a significant decrease in the frequency of polyfunctional CD8^+^ T cells in patients with severe disease and an increase in inhibitory receptors on CD8^+^ T cells from CCPs (Lasso, P, et al, manuscript in preparation). However, it has been shown in *T. cruzi*-infected mice that perforin-producing cells may contribute to cardiomyocyte lesions and heart dysfunction during chronic *T. cruzi* infection [31]. We observed an increased frequency of T_TE_ cells and a higher frequency of perforin-producing cells in CCPs with severe forms of the disease (Lasso, P, et al, manuscript in preparation). Because T_TE_ cells have a greater cytotoxic capacity than early differentiated cells, we suggest that a high frequency of T_TE_ cells may be associated with the heart damage observed in CCPs due to a high frequency of perforin-producing cells observed in these patients.

Based on functional CD8^+^ T cell responses, it was previously reported that *T. cruzi*-specific CD8^+^ T cells from chronic *T. cruzi*-infected individuals display a functional profile with T cells secreting IFN-γ alone as the predominant pattern and a very low prevalence of single IL-2-secreting cells [32]. However, in *T. cruzi*-infected mice, parasite persistence has been associated with the phenotype of CD8^+^ T cells because the absence of antigenic load is correlated with an increased frequency of early differentiated cells [33]. The findings of the present study with parasite persistence in CCPs could be associated with the changes observed both in the frequencies and functional activities of CD8^+^ T cell subsets, as has been described for other chronic infections [34], [35].

In chronic *T. cruzi* infection, the parasitaemia load is low [36]. In our study, parasite DNA was detectable in peripheral blood in 40% of patients. This result is similar to findings in other studies that identified parasitaemia in CCPs, where detection by PCR is between 40–50% of confirmed positive samples [37], [38]. However, in positive blood donors detected by other tests for *T. cruzi* infection, it has been reported that PCR is positive in only 13% [39]. In addition, a lack of association between blood-based detection of parasite DNA and cardiac damage has been reported [40]. We find parasite DNA even in the most severe forms of disease but the lack of association is most likely due to low parasitism in blood and tissue, the anatomical location of parasites in CCPs and parasite variability [40].

In summary, IFN-γ-, TNF-α- and IL-2-producing *T. cruzi*-specific CD8^+^ T_SCM_ cells and polyfunctionality were not detectable in CCPs with severe forms of disease. In the context of chronic *T. cruzi* infection, we hypothesized that CCPs with mild disease would be able to control infection and prevent the progression of the disease via T_SCM_ cells and polyfunctional cells at early differentiation stages. For unknown reasons, some infected individuals lost the ability to regenerate antigen-specific CD8^+^ T_SCM_ cells to create enough polyfunctional cells to control the infection. Memory cells can differentiate into T_TE_ cells with only one function and probably with high expression of inhibitory receptors. Overall, these findings related to changes in the distribution of CD8^+^ T cell subsets, functional activity of CD8^+^ T cells and parasite persistence in CCPs may be associated with the immune response to and outcome of CD. However, it is still important to evaluate the role of CD8^+^ T_SCM_ cells in parasite control of the infection.

## Supporting Information

S1 Fig **Trend analysis of total CD8^+^ T cell subsets from CCPs.** Frequency of CD8^+^ T_SCM_, T_CM_, T_TM_, T_EM_ and T_TE_ cells from CCPs with different degrees of disease severity. The *p* values were calculated using a simple linear regression. CCPs were grouped according to the disease severity as described in Materials and Methods (A = 10, B = 8, C = 9 and D = 5).
(TIFF)

S2 Fig **Correlation analysis of absolute numbers of T_SCM_ and T_TE_ cells from CCPs.** Correlations between the absolute numbers of CD8^+^ T_SCM_ cells and CD8^+^ T_TE_ cells from CCPs were calculated with Spearman's rank correlation coefficient. CCPs were grouped according to the disease severity as described in Materials and Methods (A = 10, B = 8, C = 9 and D = 5).
(TIFF)

S3 Fig **Functional activity profiles of** ***T. cruzi*** **-specific CD8^+^ T cell subsets from CCPs.** (**A**) Frequencies of CD8^+^ T_SCM_, T_CM_, T_EM_ and T_TE_ cells with one, two and three functions among CD8^+^ T cells stimulated with Staphylococcal enterotoxin B (SEB). (**B**) Frequencies of CD8^+^ T_SCM_, T_CM_, T_EM_ and T_TE_ cells with one, two and three functions among CD8^+^ T cells stimulated with parasite lysate. Box and whiskers indicate the median frequency and range of the CD8^+^ T cell subsets (25–75 percentile). The *p* values were calculated using a Wilcoxon signed-rank test (*p<0.05, **p<0.01, ***p<0.001, ****p<0.0001).
(TIFF)
