# Supplementary figures and images for: Low Frequency of Circulating CD8+ T Stem Cell Memory Cells in Chronic Chagasic Patients with Severe Forms of the Disease
Source: PLoS Negl Trop Dis. 2015 Jan 8;9(1):e3432. doi: 10.1371/journal.pntd.0003432 (PMC4287481; doi:10.1371/journal.pntd.0003432)

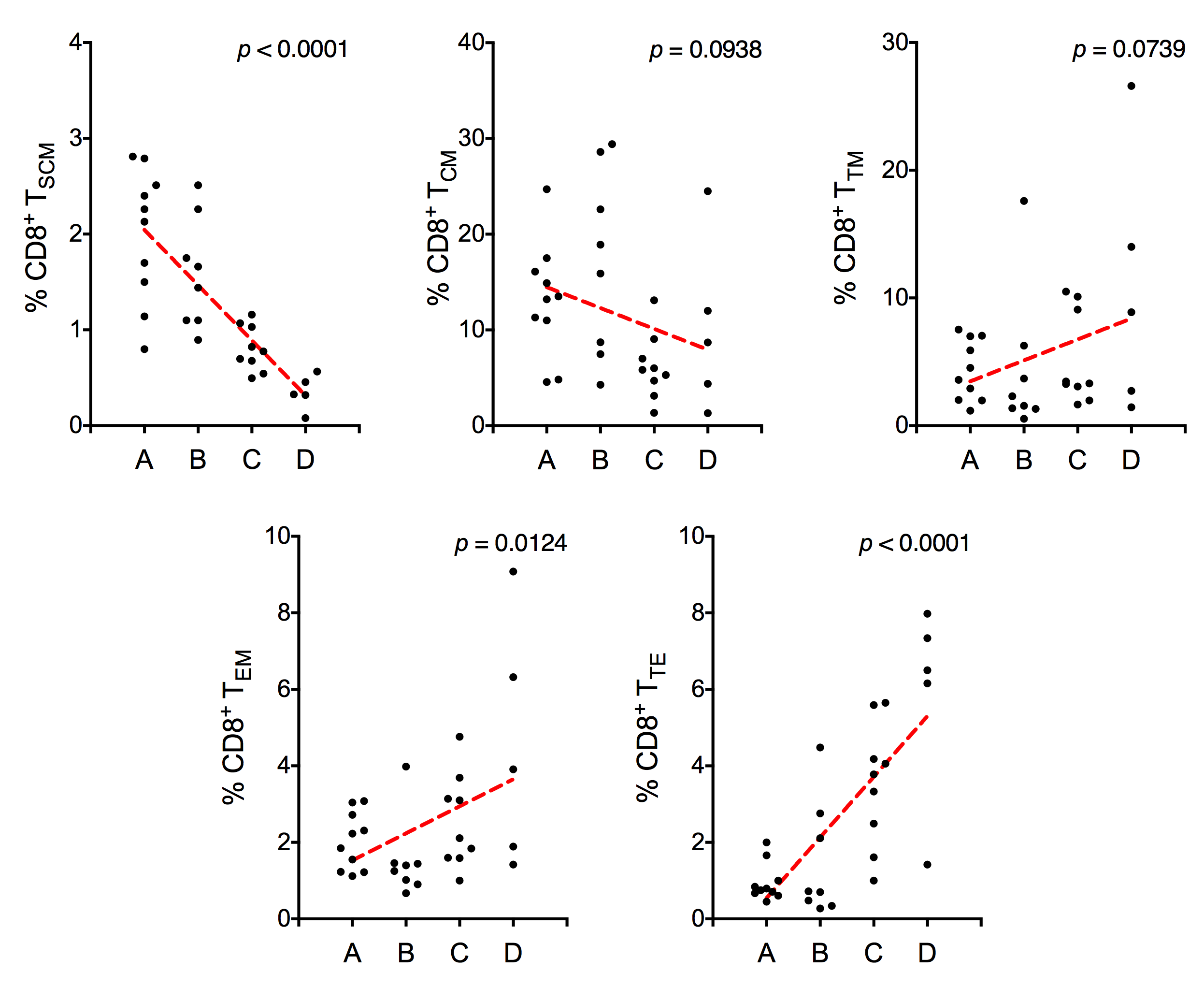

Supplement: S1 Fig — Trend analysis of total CD8+ T cell subsets from CCPs. Frequency of CD8+ TSCM, TCM, TTM, TEM and TTE cells from CCPs with different degrees of disease severity. The p values were calculated using a simple linear regression. CCPs were grouped according to the disease severity as described in Materials and Methods (A = 10, B = 8, C = 9 and D = 5). (TIFF) [file pntd.0003432.s001.tiff]

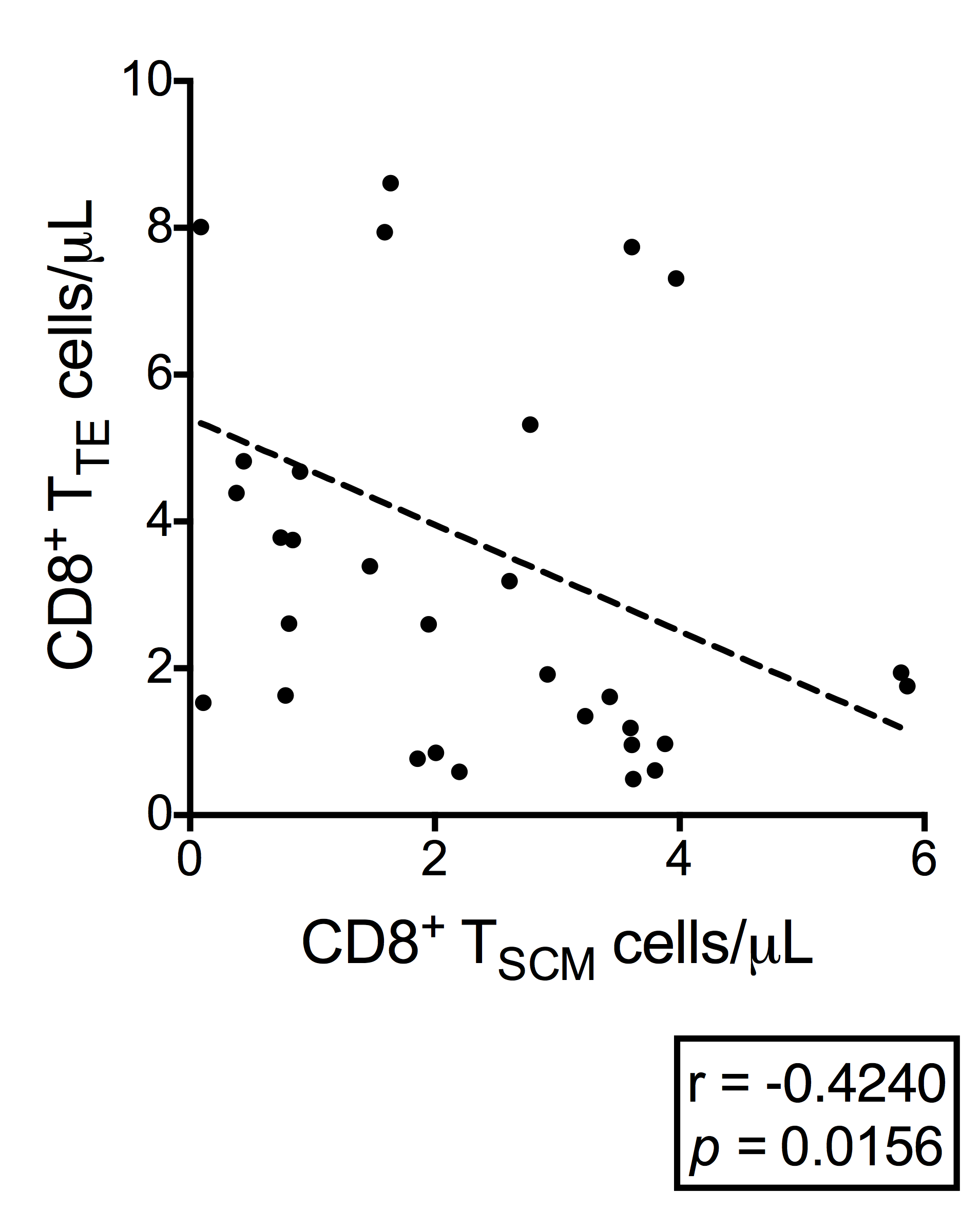

Supplement: S2 Fig — Correlation analysis of absolute numbers of TSCM and TTE cells from CCPs. Correlations between the absolute numbers of CD8+ TSCM cells and CD8+ TTE cells from CCPs were calculated with Spearman's rank correlation coefficient. CCPs were grouped according to the disease severity as described in Materials and Methods (A = 10, B = 8, C = 9 and D = 5). (TIFF) [file pntd.0003432.s002.tiff]

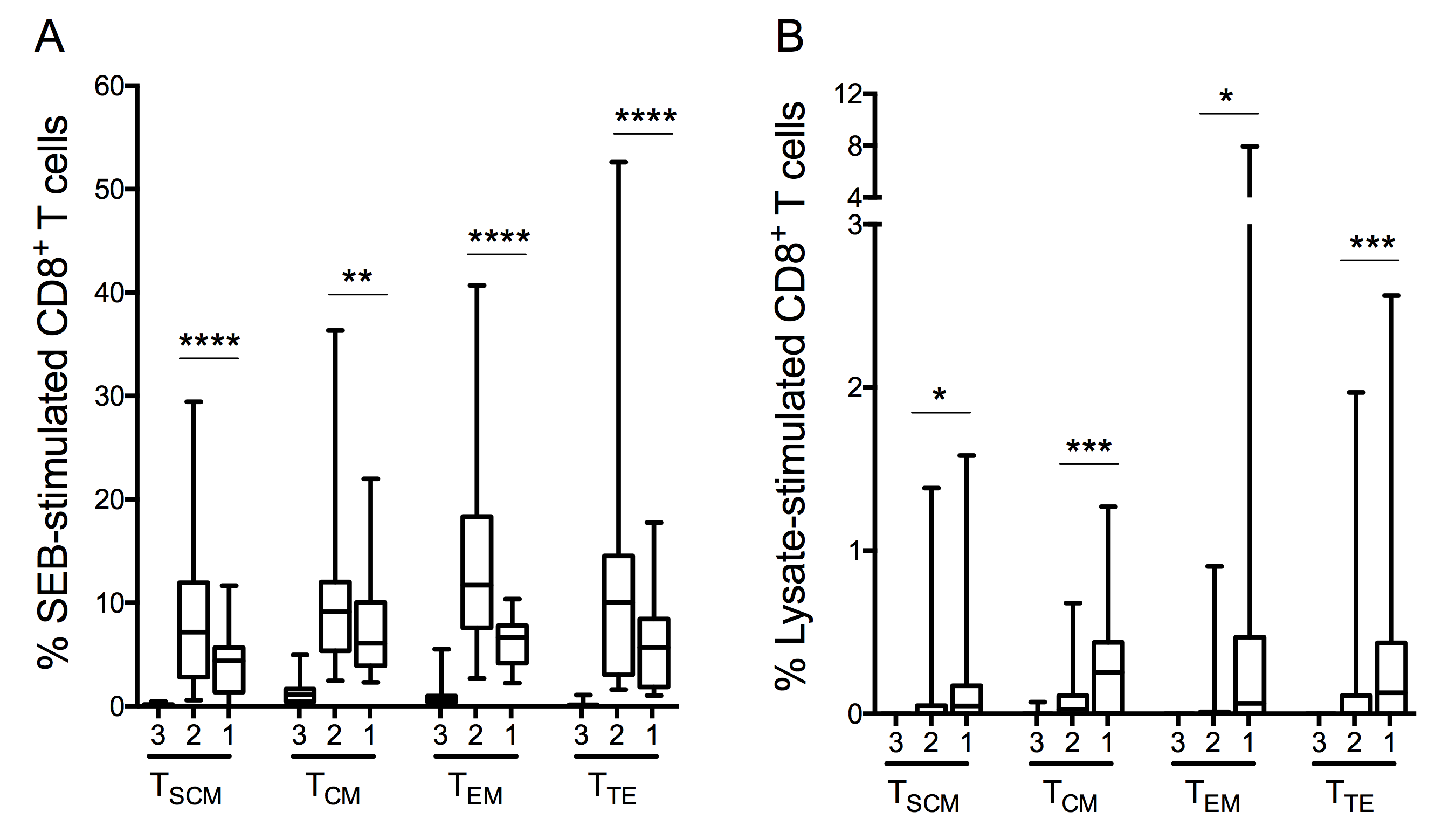

Supplement: S3 Fig — Functional activity profiles of T. cruzi -specific CD8+ T cell subsets from CCPs. (A) Frequencies of CD8+ TSCM, TCM, TEM and TTE cells with one, two and three functions among CD8+ T cells stimulated with Staphylococcal enterotoxin B (SEB). (B) Frequencies of CD8+ TSCM, TCM, TEM and TTE cells with one, two and three functions among CD8+ T cells stimulated with parasite lysate. Box and whiskers indicate the median frequency and range of the CD8+ T cell subsets (25–75 percentile). The p values were calculated using a Wilcoxon signed-rank test (*p<0.05, **p<0.01, ***p<0.001, ****p<0.0001). (TIFF) [file pntd.0003432.s003.tiff]
